# Effect of curry leaves in lowering blood pressure among hypertensive Indian patients

**DOI:** 10.6026/973206300191020

**Published:** 2023-10-31

**Authors:** R Gopal, R Ambiha, N Sivasubramanian, Patel Vidh Bhupendrabhai, Solanki Itishaben Itishaben Girishbhai, Solanki Nilam Govindbhai, Sutariya Darshan Narendrabhai, Suthar Niyatiben Jigneshkumar, Vaghela Artiben Rameshbhai

**Affiliations:** 1 Nootan College of Nursing, Sankalchand Patel University, Visnagar, Gujarat-384315, India

**Keywords:** Curry leaves, blood pressure, hypertension, rural area

## Abstract

Hypertension, commonly known as high or rising blood pressure, is characterized by a consistently elevated blood arterial pressure. It is a significant medical condition called hypertension raises your risk of developing problems with your heart, brain, kidneys, and other organs. Curry leaves work well as a treatment for high blood pressure. Curry leaves are thought to have a potent therapeutic impact for the treatment of high blood pressure since they are low in salt and high in potassium. Therefore, it is of interest to reduce the level of blood pressure among adults in rural area with the help of curry leaves. According to the statistical analysis, the experimental group's post-test mean score for hypertension was 155 with a standard deviation of 13.892 while the control group's post-test mean score was 162 with a standard deviation of 17.20. The estimated unpaired value of t = 11.07 was deemed to be statistically significant at the p 0.05 level and the mean difference was 7. As a result, the null hypothesis was rejected and the research hypothesis was accepted. Giving Curry leaf powder to patients with hypertension as a consequence was an effective intervention.

## Background:

Your chance of developing heart, brain, kidney, and other problems increases because of a serious medical disease called hypertension. With each beat, the heart pumps blood into the vessels. Blood pressure is caused by the heart's action of pushing blood against artery walls. The most common preventative risk factor for cardiovascular disease (CVD) are coronary heart disease, heart failure, stroke, myocardial infarction, atrial fibrillation, and peripheral artery disease), cognitive decline, and chronic kidney disease (CKD). Additionally, it is the primary global cause of mortality and disability. It's important to consider a person's predicted atherosclerotic CVD (ASCVD) risk while treating hypertension rather than just their blood pressure because those with a high CVD risk benefit the most from taking blood pressure-lowering drugs [1]. Worldwide, hypertension significantly contributes to cardiovascular disease and deaths, especially in low- and middle-income (LMIC) countries. In 2019, 1.28 billion people between the ages of 30 and 79 are expected to be affected by hypertension, with prevalence rates of 32% for women and 34% for men. Increased exposure is caused by the age of the population as well as lifestyle risk factors such bad diets (high salt and low potassium consumption) and inactivity. The number of DALYs linked to hypertension rose from 21 million in 1990 to 39 million in 2016 (+89%) in India as well [2]. Cardiovascular morbidity and mortality are increased by hypertension by a factor of two to four. The degree of systolic or diastolic blood pressure elevation at any age, in either sex, is inversely correlated with risk. More so than the specifics of blood pressure elevation, the risk is significantly influenced by frequently present risk factors. Patients with hypertension who also have a high total/high-density lipoprotein (HDL)-cholesterol ratio, impaired glucose tolerance, high fibrinogen levels, abnormal electrocardiographic (ECG) results, and who smoke cigarettes are at a higher risk of developing coronary heart disease. Impending cardiovascular consequences are characterized by organ involvement evidence, such as left ventricular function. ECG-LVH exhibits the same clinical characteristics as myocardial infarction and predisposes patients at the same rate to sudden death, infarction, cardiac failure, and stroke [3]. The most frequently reported symptoms were headache, dizziness, fever, and mood changes (poor mood, frustration, and irritability). Chest tightness, palpitations, back discomfort, constipation, and impaired vision are further reactions. Typically, people with un-accelerated undocumented hypertension are not anticipated to experience these symptoms. We cannot automatically conclude that the existence of these symptoms is a result of high blood pressure. Potentially detrimental is the patient's notion that their symptoms are connected to hypertension. Antihypertensive medications are used to address symptoms including palpitations, dizziness, and poor mood that may otherwise urge a patient to seek medical attention. A patient was using additional drugs to address her debilitating headaches. Many patients believe that having low blood pressure will make them feel better, which raises the possibility of low compliance due to treatment's perceived inefficacy [4]. Lowering blood pressure can reduce the risk of cardiovascular disease and can be in mild cases; lifestyle intervention is achieved and should be the first approach treat blood pressure in all cases. This includes dietary intervention, body weight smoking cessation and physical activity. Complex hypertension management should focus not only on lowering blood pressure, but also on lowering blood pressure cardiovascular risk through lifestyle intervention, lipid control, smoking cessation and regular exercise [5]. Systolic blood pressure in particular has been associated with a number of detrimental clinical outcomes, such as stroke, heart failure, myocardial infarction, renal failure, peripheral vascular disease, retinopathy, dementia, and early mortality. Although not always, pressure-related target-organ harm such as left ventricular hypertrophy, renal insufficiency, and proteinuria precedes these adverse clinical consequences [6]. Hypertension can be treated with Diuretics, adrenergic inhibitors, direct vasodilators, calcium channel blockers, ACE - inhibitors, angiotensin-2 blockers are reduce the high blood pressure. Other lifestyle modification also fruitful for reducing high blood pressure includes, Weight reduction, Adopt DASH eating plan, Dietary sodium reduction, Physical activity and Moderation of alcohol consumption [7]. Asian nations have traditionally employed Murraya koenigii (curry leaves) to treat conditions including diabetes and hypertension. Using a carrageenan-induced paw edema technique, the anti-inflammatory efficacy of Murraya koenigii leaf extracts was assessed in rats. When compared to the usual medication, diclofenac, ethanolic extract shows a substantial anti-inflammatory action three hours after carrageenan administration. [8]. Three types of curry leaves in the Rutaceae family are *Murraya koenigii* (*M. koenigii*), *Micromelum minutum* (*M. minutum*), and *Clausena indica* (*C. indica*). They have been extensively utilized around the world in Ayurvedic medicine for the treatment and prevention of numerous illnesses. The three curry leaf species have powerful pharmacological and biological effects, including antioxidant, anti-diabetic, anti-inflammatory, and anticancer activity, which are supported by earlier research. These plants' various sections, including their leaves, seeds, flowers, and fruit, contain substances that regulate a variety of biological processes [9]. Curry leaves are also helpful for lowering cholesterol, enhancing digestion, protecting the liver, promoting weight loss, improving blood circulation, and accelerating hair development. Curry leaves efficiently lower high blood pressure, acting similarly to the blood pressure medication amlodipine [10]. Therefore, it is of interest to document the effect of curry leaves in lowering blood pressure among hypertensive Indian patients

## Methodology:

The current study's objective was to reduce the level of blood pressure among adults in rural area with the help of curry leaves. For the study, a quasi-experimental study with pre-test and post-test design was employed. For this investigation, an experimental group and control group pre-test and post-test design was chosen. The information was gathered from 50 Kansa-based adults with hypertension made up the sample for this study; 25 of them were chosen to form the experimental group and 25 to form the control group. The study employed the non-probability purposive sampling method. 10 grams of curry leaves powder should be given daily in the morning and evening before meals for 30 days. A structured modified IBHI risk assessment tools was utilized for pre-test and post-test. Post test was conducted after 21 days of curry leaves administration. Descriptive and inferential statistics like mean, standard deviation, and chi square test were used to examine the data.

## Results:

In demographic characteristics the data shows that 4 (16%) of were in the age of 40-45 years, 7(28%) of adults were in the age of 46-50 years, 4(16%) of adults were in the age 51-55 years, 10(40%) of adults were in the age of 56-60 years. Majority 15(60%) of female and 10 (40%) of male, 9(36%) of adults are illiterates, 10(40%) of adults have primary education, 3(12%) of adults have Higher secondary education, 2(8%) of adults are from graduates status and 1 (4%) adult is a Post graduate. 14 (56%) of adults were in Home maker, 2(8%) adults were in private employee, 2 (8%) adults were in government employee, 7 (28%) adults were in self-employee. 7(28%) adults were eating fried foods, 4 (16%) adults were eating fast foods, 14(56%) adults were eating saturated and fat foods. 5(20) of adults were performing exercises, 20 (80%) of adults were not performing exercises. 3(12%) of adults were smoking, 4(16) of adults were alcoholic, 4(16%) of adults were use tobacco, 2(8%) of adults were using all three (smoking, alcohol, tobacco), 12(48%) of adults had none of these habits.

In clinical variables the data shows that 11(44%) of hypertensive adults had a family history of hypertension, 14 (56%) of hypertensive adults had no family history of hypertension. 2 (8%) of adults had hypertension for less than a year, 4(16%) of adults had hypertension for 1-2 years, 8(32%) of adults had hypertension for 3-5 years, 11(44%) of adults had hypertension for more than five years. 7(28%) of adults had symptoms of Headache , 2(8%) of adults had symptoms of Dizziness, 2(8%) of adults had symptoms of Nausea and vomiting, 3(12%) of adults had symptoms of Palpitation, 11(44%) of adults had no any symptoms of hypertension. 15(60%) of adults were on treatment for hypertension, 10(40%) adults were not taken treatment. 6 (24%) adults had complications of vision loss due to hypertension, 2(8%) of adults had complications of heart failure, 2(8%) of adults had complications of kidney failure, 3(12%) of adults had complications of stroke, 12(48%) of adults had no any complications of hypertension.

The above Table 1 shows that Pre-test and Post- test group of experimental group and control group. In pre- test, the experimental group 9 clients were on severe hypertension, 10 clients were on mild hypertension and 6clients were on moderate hypertension. Where as in control group 9 clients were on severe hypertension, 9 clients were on mild hypertension and 7 clients were on moderate hypertension. In post-test, the experimental group 14 clients were on mild hypertension, 8clients were on moderate hypertension and 3 clients were on severe hypertension. Whereas, in control group 10 clients were on mild hypertension, 7 clients were on severe hypertension and 8 clients were on moderate hypertension.

A paired 't' test was employed to examine the significant difference between the mean scores in the experimental groups and the control group prior to and following the addition of curry leaves, as shown in Table 2Pre-test mean and SD for the experimental group was 164.4 and 18.35, respectively, and post-test mean and SD were 155 and 13.892, respectively. The pre-test mean score for the control group was 164, the SD was 17.20, and post-test mean score was 162, also with an SD of 17.20. Eventually the experimental groups' 't' test value is 9.346 and the control group's 't' test value is 4. The results of the modified IHBI risk assessment toolrevealed that there is a highly significant difference in experimental group and a significant difference in the control group. Hence the research hypotheses were accepted and null hypotheses was rejected. Table 3

According to the above table, the post-test mean score for the experimental group was 155, with a standard deviation of 13.892, while the post-test mean score for the control group was 162, with a standard deviation of 17.20, yielding a mean difference of 7, and a computed unpaired value of t = 11.07, which must be greater than 0.05 to be statistically significant. Hence the research hypotheses was accepted and null hypotheses was rejected.

The current study discovered that the level of blood pressure were significantly different between the pre and post-test. Thus, it was clear that curry leaves had a positive impact on hypertensive patients. According to the literature review by Murraya Koenigii, the experimental group received a daily supplemental dose of 10 grams of curry leaf powder chutney (including 5 grams of curry leaf powder) for a total of 60 days. Blood pressure values were taken at 0, 30, and 60 days after the experiment began. By the end of the study, the addition of curry leaves chutney had reduced both systolic blood pressure (147 mm Hg to 130 mm Hg) and diastolic blood pressure (93 mmHg to 83 mm Hg). This inventive use of curry leaves, which are widely available, can be monetized as a value-added product that promotes a healthy lifestyle by lowering blood pressure [11]. Another study found that descriptive and inferential statistical procedures, including the chi square test, f test, mean, median, SD, post hoc test, Mann Whitneys U test, and Wilcoxon signed rank test, were used to examine the data. Ayurvedic doctor's recommendations were followed when making curry leaf juice, which was given to pre hypertension patients each morning. The findings showed a significant change in diastolic blood pressure between baseline and immediately after administering curry juice (Z=4.487P=0.001*) as well as a significant difference in systolic blood pressure between baseline and immediately after delivering curry juice (Z=4.787P=0.001*). The study finds that using curry leaf juice to treat pre-hypertension patients can be done affordably [12]. The review of study was conducted in Tamilnadu. Single group pre-test-post-test design with quantitative methodology was utilized for the study. By using the purposive sampling strategy, 65 samples were chosen. Using a standardized sphygmomanometer, blood pressure levels were measured before and after the test. The study's findings indicate that the pre-test's mean scores for systolic and diastolic pressure were, respectively, 146.25 and 94.06. Systolic blood pressure in post-test II averaged 131.09 and was significant at the 0.05 level. It is assumed that a diastolic blood pressure of 79.06 indicates that there is one. The measured 't' value indicated a statistically significant difference in the blood pressure levels of the hypertension patients. As a result, providing Curry leaf powder to patients with hypertension was a successful intervention [13].

Curcuma longa, which has high curcumin content, is utilized in both food and medicine, particularly in curry dishes. Using data from the 2013 Korean National Health and Nutrition Examination Survey, the current study examined the relationship between curry consumption and blood levels of heavy metals and high blood pressure. According to the analysis's results, eating curry considerably reduced blood levels of the heavy metals Pb, Hg, and Cd, which were also strongly associated with the prevalence of HTN (P 0.001 for Pb, Hg, and Cd). Eating curry lowered the risk of HTN development as well. We recommend conducting more painstakingly planned clinical trials due to the prevalence of HTN and the link between consuming curry and having lower blood levels of heavy metals [14].

## Conclusion:

Curry leaves are inexpensive and widely available. Additionally, the value-added component of curry powder aids in the more effective utilization of curry leaves. In addition to increasing dietary contribution, acceptance, and it improves overall health by lowering blood pressure. There was a huge drop in values. Curry powder raises blood pressure. Curry leaves' chemical composition is to blame for this. Therefore, the current study supports that association. A modulating effect is had by curry powder. High blood pressure management and confirmation of some of its management

## Figures and Tables

**Table 1:** Percentage level of Modified IHBI risk assessment tools for among patients with Hypertension before and after curry leaves

	**Experimental group**				**Effect**	**Control group**				**Effect**
**Stages of Hypertension**	**Before**		**After**			**Before**		**After**		
	N	%	N	%		N	%	N	%	
Mild Hypertension	10	40	14	56	16	9	36	10	40	4
Moderate Hypertension	6	24	8	32	8	7	28	8	32	4
Severe Hypertension	9	36	3	12	24	9	36	7	28	8
Total	25	100	25	100		25	100	25	100	

**Table 2:** Comparison of the Effectiveness of Experimental group and control group on Selected modified IHBI risk assessment tool.

	**Experimental group**		**Paired 'T' test**	**Table value**	**Control group**		**Paired 'T' test**	**Table value**	**Level of Significant**
**Level of test**	**Mean**	**Std. Deviation**			**Mean**	**Std. Deviation**			
Pre test	164.4	18.35	9.346	24	164	17.2	4	24	Significant
Post test	155	13.89			162	17.2			

**Table 3:** Comparison of Post-test mean score of hypertensions among adults with curry leaves between Experimental group and Control group

**Group**		**Mean**	**Std. Deviation**	**Unpaired 't' Test**	**DF**	**Table Value**	**Sig/Non Sig**
Hypertension	Experimental	155	13.892	11.07	48	1.671	Significant
	Control	162	17.2				
